# Risk factors of pneumothorax and pneumomediastinum in COVID-19: a matched case–control study

**DOI:** 10.1186/s12879-023-08104-3

**Published:** 2023-03-07

**Authors:** Se Ju Lee, Jinnam Kim, Ki Hyun Lee, Jung Ah Lee, Chang Hyup Kim, Su Hwan Lee, Byung Jo Park, Jung Ho Kim, Jin Young Ahn, Su Jin Jeong, Nam Su Ku, Joon-Sup Yeom, Jun Yong Choi

**Affiliations:** 1 Division of Infectious Diseases, Department of Internal Medicine and AIDS Research Institute, Yonsei University College of Medicine, Seoul, Republic of Korea; 2 Division of Infectious Diseases, Department of Internal Medicine, Inha University College of Medicine, Incheon, Republic of Korea; 3 Division of Pulmonary and Critical Care Medicine, Department of Internal Medicine, Yonsei University College of Medicine, Seoul, Republic of Korea; 4 Department of Thoracic and Cardiovascular Surgery, Yonsei University College of Medicine, Seoul, Republic of Korea

**Keywords:** COVID-19, Risk factors, Pneumomediastinum, Pneumothorax

## Abstract

**Background:**

During the novel coronavirus disease-2019 pandemic, a considerable number of pneumothorax (PNX)/pneumomediastinum (PNM) associated with COVID-19 have been reported, and the incidence is higher in critically ill patients. Despite using a protective ventilation strategy, PNX/PNM still occurs in patients on invasive mechanical ventilation (IMV). This matched case–control study aims to identify the risk factors and clinical characteristics of PNX/PNM in COVID-19.

**Methods:**

This retrospective study enrolled adult patients with COVID-19, admitted to a critical care unit from March 1, 2020, to January 31, 2022. COVID-19 patients with PNX/PNM were compared, in a 1–2 ratio, to COVID-19 patients without PNX/PNM, matched for age, gender, and worst National Institute of Allergy and Infectious Diseases ordinal scale. Conditional logistic regression analysis was performed to assess the risk factors for PNX/PNM in COVID-19.

**Results:**

427 patients with COVID-19 were admitted during the period, and 24 patients were diagnosed with PNX/PNM. Body mass index (BMI) was significantly lower in the case group (22.8 kg/m^2^ and 24.7 kg/m^2^; *P* = 0.048). BMI was statistically significant risk factor for PNX/PNM in univariate conditional logistic regression analysis [odds ratio (OR), 0.85; confidence interval (CI), 0.72–0.996; *P* = 0.044]. For patients on IMV support, univariate conditional logistic regression analysis showed the statistical significance of the duration from symptom onset to intubation (OR, 1.14; CI, 1.006–1.293; *P* = 0.041).

**Conclusions:**

Higher BMI tended to show a protective effect against PNX/PNM due to COVID-19 and delayed application of IMV might be a contributive factor for this complication.

## Background

Although more than 2 years have passed since the onset of the coronavirus disease-2019 (COVID-19) pandemic, the disease is still causing problems and spreading swiftly on a global scale [1]. In addition to the high transmissibility, the high fatality rate of COVID-19 has resulted in numerous cases of critically ill patients [2]. In particular, many cases of pneumothorax (PNX)/pneumomediastinum (PNM) associated with COVID-19 have been reported, and the incidence is higher in critically ill patients [3–5].

PNX/PNM occasionally occurs as a complication in patients with pneumonia, sometimes resulting in detrimental effects, such as longer stay in the intensive care unit and higher mortality [6–8]. In particular, when PNX/PNM occurs in patients on invasive mechanical ventilation (IMV) support, the complications can be fatal, so a protective ventilation strategy is implemented in critical care to prevent these complications [9, 10].

PNX/PNM significantly affects the mortality rate of patients with COVID-19 [4, 11]. Despite the use of a protective ventilation strategy, PNX/PNM still occurs in patients with acute respiratory distress syndrome (ARDS) caused by COVID-19 [12]. Therefore, it is essential to identify risk factors for PNX/PNM and formulate countermeasures. Several studies have been conducted to determine the risk factors for PNX/PNM in COVID-19 patients [3, 13–15]. However, most previous studies have not clarified the risk factors and lack adjustment for disease severity; hence, there is a need to establish the risk factors for PNX/PNM in COVID-19. The aim of this matched case–control study was to identify the risk factors and clinical characteristics of PNX/PNM in patients with COVID-19.

## Methods

### Study design and patient population

In this retrospective study, we enrolled the patients diagnosed with COVID-19, admitted to Severance Hospital from March 1, 2020, to January 31, 2022. This hospital was running a critical care unit for critically ill COVID-19 patients in South Korea during the pandemic.

Patients were included if they met the following criteria: (1) older than 17 years, (2) diagnosed with COVID-19 and admitted to Severance Hospital, and (3) diagnosed with PNX/PNM by chest radiography or computed tomography. COVID-19 was diagnosed using real-time reverse transcriptase polymerase chain reaction (PCR) test. Patients with PNX/PNM due to iatrogenic causes were excluded.

For risk factor analysis, control subjects were obtained from the same patient population. Two control subjects were individually matched for each patient with COVID-19 according to the following characteristics: (1) age, (2) sex, and (3) the worst National Institute of Allergy and Infectious Diseases ordinal scale (NIAID-OS) score during treatment. NIAID-OS scores were categorized as follows: 1, not hospitalized, no limitations of activities; 2, not hospitalized, limitation of activities and/or requiring home oxygen; 3, hospitalized, no supplemental oxygen and no longer requires ongoing medical care; 4, hospitalized, no supplemental oxygen, but requires ongoing medical care; 5, hospitalized, requiring supplemental oxygen; 6, hospitalized, on non-invasive ventilation or high-flow oxygen devices; 7, hospitalized, on invasive mechanical ventilation or extracorporeal membrane oxygenation; and 8, death [16].

### Ethical statement

The Institutional Review Board of the Yonsei University Health System Clinical Trial Centre approved this study on 24 May 2021 (4-2021-0510). Because the study was retrospective and the data were anonymized, the IRB waived the requirement for informed consent.

### Variables and definitions

All relevant clinical and laboratory data were collected from electronic medical records. Data pertaining to the ventilator settings of the patients on IMV support and of the laboratory tests of all the patients were collected according to the index date of each patient. In the case group, for patients who did not receive IMV support, the index date was defined as the day with the highest recorded oxygen demand before the development of PNX/PNM. For those who received IMV support, the index date was set as the day with the highest recorded peak pressure before the development of PNX/PNM. In the matched-control group, for the patients who did not receive IMV support during admission, the index date was defined as the day with the highest recorded oxygen demand, and for those who received IMV support, the index date was set as the day with the highest recorded peak pressure during admission. Disease severity was classified according to the worst National Institute of Allergy and Infectious Disease Ordinal Scale during admission.

The Charlson Comorbidity Index was calculated at admission to classify patients according to overall comorbidity. The sequential organ failure assessment (SOFA) score was used to measure the severity of organ dysfunction. Superimposed pneumonia was defined as follows: (1) new or worsening infiltrates on chest radiography, (2) positive bacterial culture from the respiratory specimen or positive PCR results for other respiratory pathogens, and (3) administration of antimicrobial agents against newly identified pathogens. COVID-19-associated pulmonary aspergillosis was defined as proven or probable invasive aspergillosis based on the definition proposed by the EORTC/MSGERC ICU Working Group [17].

The objective of this study was identifying risk factors associated with PNX/PNM for patients with COVID-19.

### Statistical analysis

Differences in patient characteristics and outcomes were assessed between the two groups using the chi-squared test or Fisher’s exact test for categorical variables and the Wilcoxon rank-sum test for continuous variables according to results from a Shapiro–Wilk test. Conditional logistic regression analysis was performed to assess the risk factors for PNX/PNM in COVID-19 patients. Variables with *P* < 0.1 in univariate analyses and with clinical relevance were entered into a multivariable model. Statistical significance was set at *P* < 0.05. All statistical analyses were performed using R V.4.0.5 (The R Foundation for Statistical Computing, Vienna, Austria).

## Results

### Number of cases with PNX/PNM

**Fig. 1:**
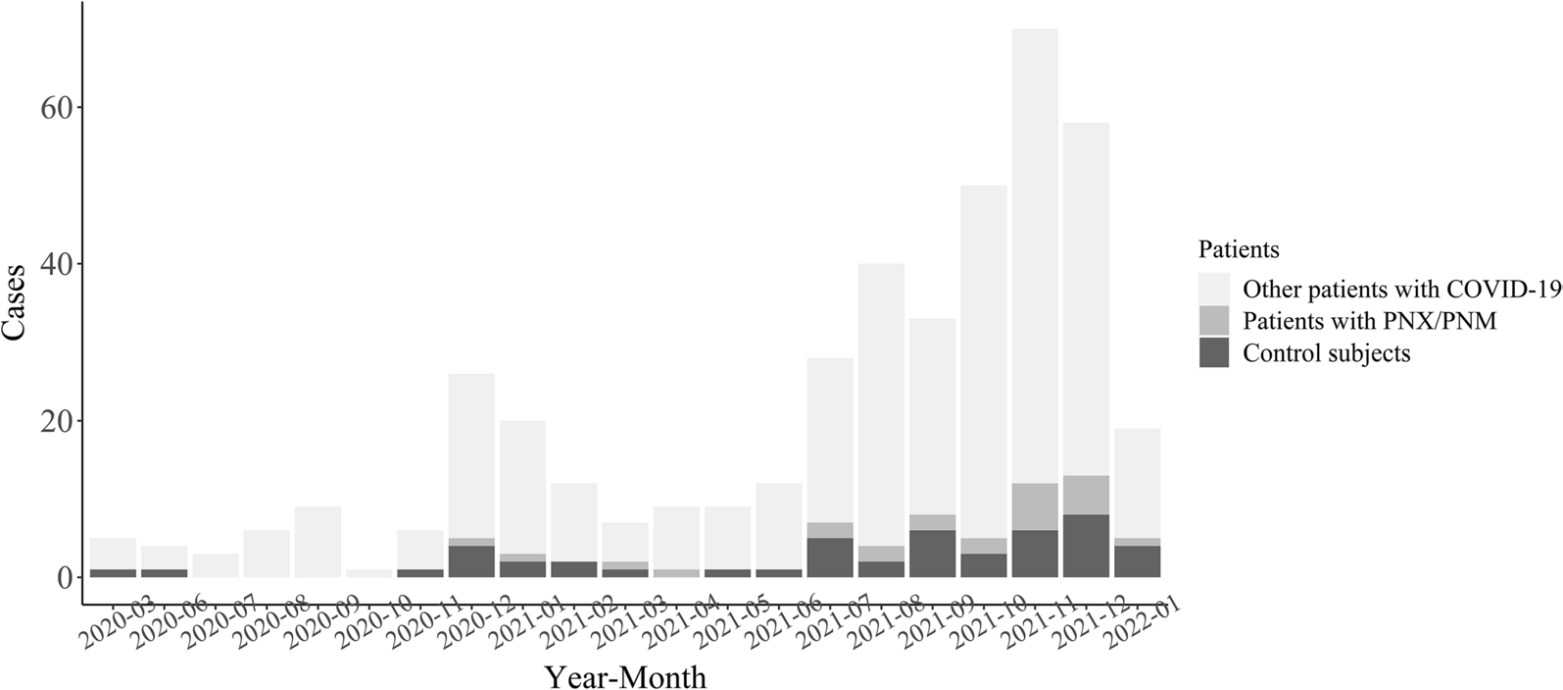
The number of COVID-19 patients and PNX/PNM cases by month during the study period. No patients were admitted to the critical unit from April to May 2020; COVID-19, coronavirus disease-2019; PNX/PNM, pneumothorax/pneumomediastinum

Figure 1 shows the number of COVID-19 patients admitted to this critical care center each month during the study period. Patients with PNX/PNM were not concentrated within a specific period and their monthly concentration tended to be in proportion to the total number of COVID-19 patients per month. A total of 427 patients with COVID-19 were admitted during the study period. Of these, 24 were diagnosed with PNX/PNM (case group). When classified by the NIAID-OS score, 0 of 41 patients (0%) with an NIAID-OS score of 4 points, 2 of 101 patients (2.0%) with an NIAID-OS score of 5, 4 of 133 patients (3.0%) with a score 6 points, and 18 of 152 patients (11.8%) with a score of 7–8 points had PNX/PNM during treatment. PNX/PNM developed during treatment in 18 of 128 (14.1%) patients who were on IMV support and in 6 of 299 (2.0%) patients who were not on IMV support. Forty-eight patients were matched with the 24 case patients and enrolled in the control group.

### Clinical characteristics and treatment outcomes

On comparing the characteristics of the patients in the two groups, no statistical differences were found in age, sex, and NIAID-OS scores (Table 1). Body mass index (BMI) was significantly lower in the case group (22.4 kg/m^2^; interquartile range [IQR], 20.4–24.6 and 24.5 kg/m^2^; IQR, 22.0–27.3; P = 0.033). Four patients (16.7%) in the case group and one patient (2.1%) in the control group had asthma, showing a statistically significant difference (*P* = 0.039). There were no statistically significant differences in smoking status or medication used for COVID-19, such as dexamethasone (91.7% and 100%; *P* = 0.128), remdesivir (83.3% and 91.7%; *P* = 0.3), and other immunomodulatory agents. The rates of super-imposed pneumonia caused by pathogens other than SARS-CoV-2 and there was no difference in the PaO_2_/FiO_2_ ratios for severity assessment (156.3; IQR, 140.9–179.4 and 141.7; IQR, 110.0–196.4; *P* = 0.674). Regarding the laboratory tests performed on the index date, international normalized ratios and ferritin levels were higher in the control group, but the SOFA scores showed no significant difference (6.5 points; IQR, 2.0–8.0 and 5.0 points; IQR, 2.0–8.0; *P* = 0.99). As patients with the worst NIAID-OS were matched, both groups showed similar in-hospital mortality rates (41.7%); however, the patients in the case group tended to have longer hospital stays.

**Table 1:** Clinical characteristics and outcomes of the study population

	PNX/PNM (n = 24)	Control group (n = 48)	*P* value
Age, y	64.0 (60.0–74.0)	66.0 (56.5–74.0)	0.952
Sex, male	21 (87.5)	42 (87.5)	> 0.99
BMI, kg/m^2^	22.4 (20.4–24.6)	24.5 (22.0–27.3)	0.033
NIAID—Ordinal scale			
5	2 (8.3)	4 (8.3)	> 0.99
6	4 (16.7)	8 (16.7)	
7	8 (33.3)	16 (33.3)	
8	10 (41.7)	20 (41.7)	
Prone position during treatment	14 (58.3)	21 (43.8)	0.359
Hypertension	15 (62.5)	27 (56.2)	0.800
Diabetes mellitus	8 (33.3)	20 (41.7)	0.669
Coronary artery disease	7 (29.2)	6 (12.5)	0.108
Heart failure	0	4 (8.3)	0.294
Cardiac arrhythmia	1 (4.2)	7 (14.6)	0.255
COPD	1 (4.2)	2 (4.2)	> 0.99
Asthma	4 (16.7)	1 (2.1)	0.039
Interstitial lung disease	0	1 (2.1)	> 0.99
Chronic kidney disease	1 (4.2)	6 (12.5)	0.412
Cerebrovascular accident	0	4 (8.3)	0.294
Solid cancer	3 (12.5)	9 (18.8)	0.739
Hematologic malignancy	2 (8.3)	1 (2.1)	0.256
Solid organ transplant	1 (4.2)	3 (6.2)	> 0.99
Charlson comorbidity index	3.0 (2.0–4.0)	3.0 (2.0–5.0)	0.976
Smoking			
Current	0 (0.0)	2 (4.2)	0.542
Previous	8 (33.3)	13 (27.1)	
Never	16 (66.7)	33 (68.8)	
COVID-19 treatment			
Steroid	22 (91.7)	48 (100.0)	0.128
High-dose steroid (higher than dexamethasone 6 mg)	16 (66.7)	36 (75.0)	0.323
Remdesivir	20 (83.3)	44 (91.7)	0.300
2nd immunomodulatory agents			
Baricitinib	1 (4.2)	2 (4.2)	0.562
Tocilizumab	12 (50.0)	26 (54.2)	
Superimposed pneumonia	15 (62.5)	22 (45.8)	
Community-acquired pneumonia	1 (4.2)	1 (2.1)	> 0.99
Hospital-acquired pneumonia	13 (54.2)	19 (39.6)	0.356
COVID-19 associated pulmonary aspergillosis	1 (4.2)	5 (10.4)	0.651
White blood cell, 10^3^/μL	11.3 (7.5–16.2)	9.9 (5.6–16.3)	0.427
Lymphocyte, 10^3^/μL	0.7 (0.4–1.1)	0.6 (0.3–0.9)	0.272
Hemoglobin, g/dL	10.7 (8.6–12.0)	11.4 (8.9–13.4)	0.249
Platelet count, 10^3^/μL	190.5 (139.5–296.0)	160.0 (94.0–268.5)	0.219
International normalized ratio	1.0 (0.9–1.1)	1.1 (1.0–1.2)	0.001
D-dimer, ng/mL	548.0 (325.5–2019.5)	960.0 (402.0–3554.0)	0.111
Aspartate aminotransferase, IU/L	33.0 (23.0–50.5)	43.0 (31.0–66.5)	0.068
Alanine aminotransferase, IU/L	44.5 (24.5–63.5)	33.0 (21.5–59.5)	0.345
Total bilirubin, mg/dL	0.6 (0.4–0.8)	0.6 (0.5–1.0)	0.431
Blood urea nitrogen, mg/dL	25.0 (17.4–30.4)	26.5 (20.7–36.8)	0.330
Creatinine, mg/dL	0.6 (0.5–0.9)	0.7 (0.6–1.0)	0.164
Lactate dehydrogenase, IU/L	396.0 (303.5–484.5)	441.0 (355.5–588.5)	0.063
Ferritin, ng/mL	593.3 (240.7–927.6)	886.8 (563.4–1486.5)	0.013
C-reactive protein, mg/L	18.1 (2.2–71.0)	52.8 (16.9–115.0)	0.057
Procalcitonin, ng/mL	0.1 (0.0–0.3)	0.2 (0.1–1.0)	0.077
Arterial lactate, mmol/L	1.5 (1.0–2.4)	1.7 (1.4–2.2)	0.487
Plasma interleukin 6, pg/mL	108.0 (72.7–383.0)	89.0 (34.4–592.0)	0.557
SOFA score	6.5 (2.0–8.0)	5.0 (2.0–8.0)	0.990
PaO_2_/FiO_2_ ratio	156.3 (140.9–179.4)	141.7 (110.0–196.4)	0.674
In-hospital mortality	10 (41.7)	20 (41.7)	> 0.99
From symptom onset to hospitalization date, d	4.0 (3.0–5.5)	3.5 (2.0–7.0)	0.805
Length of stay, d	33.0 (17.5–63.0)	20.5 (13.0–31.0)	0.061

Values are count (%) for categorical variables and mean ± standard deviation or median

(Interquartile range) for continuous variables

*BMI* body mass index, *COPD* chronic obstructive pulmonary disease, *ECMO* extracorporeal Membrane oxygenation, *FiO*_*2*_ fraction of inspired oxygen, *NIAID* National Institute of Allergy and Infectious Diseases, *SOFA* sequential organ failure assessment, *PNX/PNM* pneumothorax/pneumomediastinum

Table 2 shows the data of patients who were on IMV support. There were no statistically significant differences between the two groups in the frequency of ventilator-associated pneumonia, medications used for COVID-19 treatment, rate of successful weaning from IMV, and ventilator setting. However, the duration from symptom onset to intubation (13 days; IQR, 9–18 and 9.5 days; IQR, 4–13.5; *P* = 0.032), days on IMV support (30 days; IQR, 15–74 and 17.5 days; IQR, 10.5–30; *P* = 0.083), and length of hospital stay (37.5 days; IQR, 22–74 and 23.5 days; IQR, 18–31.5; *P* = 0.052) tended to be longer in the case group.

**Table 2:** Clinical characteristics and outcomes of the patients with IMV

	PNX/PNM (n = 18)	Control group (n = 36)	*P* value
Age, y	66.6 ± 11.1	66.6 ± 11.0	0.993
Sex, male	15 (83.3)	30 (83.3)	> 0.99
Prone position during treatment	12 (66.7)	14 (38.9)	0.102
Neuro-muscular blocker use	11 (61.1)	17 (47.2)	0.500
Ventilator associated pneumonia	12 (66.7)	15 (41.7)	0.149
COVID-19 treatment			
Steroid	17 (94.4)	36 (100.0)	0.721
High-dose steroid (higher than dexamethasone 6 mg)	14 (77.8)	31 (86.1)	0.339
Remdesivir	16 (88.9)	33 (91.7)	0.344
2nd immunomodulatory agents			
Baricitinib	0	1 (2.8)	0.472
Tocilizumab	10 (55.6)	21 (58.3)	
From symptom onset to hospitalization date, d	4.0 (3.0–6.0)	3.5 (2.0–7.0)	0.663
From symptom onset to intubation date, d	13.0 (9.0–18.0)	9.5 (4.0–13.5)	0.032
Ventilator mode			
APV-CMV	2 (11.1)	4 (11.1)	0.668
Pressure-controlled	10 (55.6)	24 (66.7)	
Volume-controlled	6 (33.3)	8 (22.2)	
Tidal volume, mL	416.0 (345.0–467.0)	430.5 (401.5–459.0)	0.627
TV/Ideal body weight, mL/kg	6.8 (5.3–7.3)	6.7 (6.1–7.4)	0.472
PEEP, cmH_2_O	9.5 (8.0–12.0)	10.0 (9.0–12.0)	0.271
Peak pressure, cmH_2_O	33.5 (30.0–38.0)	31.0 (27.0–35.0)	0.282
Dynamic driving pressure, cmH_2_O	23.5 (17.0–28.0)	20.0 (17.0–24.0)	0.232
Minute ventilation, L/min	8.6 (7.4–11.5)	10.4 (8.6–12.1)	0.271
PaO_2_/FiO_2_ ratio	161.4 (141.8–197.0)	118.9 (94.2–191.1)	0.191
Arterial blood gas analysis			
pH	7.4 (7.3–7.4)	7.4 (7.3–7.4)	0.790
PaCO_2_	39.0 (35.8–53.3)	41.7 (36.9–54.1)	0.607
pO_2_	82.2 (75.5–126.9)	89.3 (72.5–111.0)	0.435
In-hospital mortality	10 (55.6)	20 (55.6)	> 0.99
Weaning from mechanical ventilation	5 (27.8)	14 (38.9)	0.614
Ventilator days, d	30.0 (15.0–74.0)	17.5 (10.5–30.0)	0.083
Length of stay, d	37.5 (22.0–74.0)	23.5 (18.0–31.5)	0.052

Values are count (%) for categorical variables and mean ± standard deviation or median

(Interquartile range) for continuous variables

*APV-CMV* adaptive pressure ventilation—controlled mechanical ventilation, *FiO*_*2*_ fraction of inspired oxygen, *IMV* invasive mechanical ventilation, *PEEP* positive end-expiratory pressure, *PNX/PNM* pneumothorax/pneumomediastinum, *TV* tidal volume

The mean duration from the onset of COVID-19 symptoms to the diagnosis of PNX/PNM was 24.5 days (IQR, 18.5–35.25 days) (Table 3). The median number of days from intubation to diagnosis of PNX/PNM was 16.5 days (IQR, 9–28.5 days). Five patients developed PNM without PNX. Among the 19 patients with PNX, 13 (68.42%) developed PNX in the right lung; chest drainage was performed in most cases (84.21%); PNX/PNM had resolved in 15 patients (62.5%) and persisted in 9 patients (37.5%). Recurrence of PNX during hospital stay was reported in 2 patients (10.53%).

**Table 3:** Characteristics and outcome of PNX/PNM

	PNX/PNM (n = 24)
Days from symptom onset to PNX/PNM	24.5 (18.5–35.25)
Days from intubation to PNX/PNM	16.5 (9–28.5)
Pneumomediastinum with pneumothorax	5
Pneumomediastinum without pneumothorax	5
Pneumothorax	19
Right	13 (68.42)
Left	3 (15.79)
Both	3 (15.79)
Pneumothorax management	
Chest drainage	16 (84.21)
Observation	3 (15.79)
Outcome	
Resolved	15 (62.5)
Remaining	9 (37.5)
Recurrence of PNX	2 (10.53)

Values are count (%) for categorical variables and median (Interquartile range) for continuous variables. PNX/PNM, pneumothorax/pneumomediastinum

### Risk factors for PNX/PNM in COVID-19

Conditional logistic regression was performed to investigate the risk factors for PNX/PNM in COVID-19 patients. BMI was statistically significant in the univariate analysis (odds ratio (OR), 0.85; confidence interval (CI), 0.723–0.996; *P* = 0.044), but not in the multivariable analysis (OR, 0.87; CI, 0.736–1.020; *P* = 0.086) (Table 4). For patients on IMV support, the duration from symptom onset to intubation was statistically significant in the univariate conditional logistic regression analysis (OR, 1.14; CI, 1.006–1.293; *P* = 0.041).

**Table 4:** Risk factors analysis for PNX/PNM in COVID-19

	Univariate analysis			Multivariable analysis		
	OR	95% CI	*P* value	OR	95% CI	*P* value
BMI	0.85	0.723–0.996	0.044	0.87	0.736–1.020	0.086
Asthma	8.00	0.894–71.575	0.063	6.01	0.647–55.858	0.115

*COVID-19* coronavirus disease-2019, *BMI* body mass index, *CI* confidence interval, *OR* odds ratio, *PNX/PNM* pneumothorax/pneumomediastinum

## Discussion

Previous studies have reported PNX/PNM as a complication and a risk factor for mortality in patients with COVID-19, especially its increasing incidence with increasing severity of COVID-19 [3–5]. Therefore, we attempted to identify the additional risk factors for PNX/PNM in COVID-19 in this matched case–control study. BMI showed statistical significance in the univariate analysis (OR, 0.85; CI, 0.723–0.996; *P* = 0.044). Emphysema-like lung changes were noted in individuals with lower BMI, and these changes might reflect a possible association between lower BMI and PNX/PNM among COVID-19 patients [18]. Although the association between BMI and the risk of spontaneous PNX remains controversial, several reports have highlighted a significant association between low BMI and spontaneous PNX [19]: People with low BMI might have unbalanced physical development, increasing negative chest pressure and increasing the risk of bulla formation and PNX [19]. Deficiencies in nutrition associated with low BMI might lead to deficiency in α1-antitrypsin, and α1-antitrypsin deficiency could promote damage of the bronchial wall [20]. To our knowledge, this study is the first to suggest that a low BMI is associated with PNX/PNM caused by COVID-19.

Underlying lung diseases such as asthma, chronic obstructive pulmonary disease, and interstitial lung disease are known risk factors for PNX/PNM [6, 7]. In our study, the proportion of asthma was significantly different between the two groups (16.7% and 2.1%; *P* = 0.039). However, the conditional logistic regression analysis did not reveal asthma as a risk factor for PNX/PNM, similar to the results of previous studies on PNX/PNM in patients with COVID-19 [3–5, 13, 14]. Tetaj et al. also reported no difference in the rates of COPD between the PNX/PNM and non-PNX/PNM groups [5]. Hence, identifying additional risk factors other than the severity of COVID-19 would improve the management of patients with COVID-19.

On comparing the patients with IMV in the study population, previously known risk factors for barotrauma, such as peak pressure, were not found to be significantly different between the two groups [11, 15]. Since a protective ventilation strategy has been established for patients with ARDS, we checked the tidal volume per ideal body weight to confirm the use of the protective ventilation strategy in the study patients [10] and found that the patients in our study were managed without significant deviations from the protective ventilation strategy (6.8 mL/kg; IQR, 5.3–7.3 and 6.7 mL/kg; IQR, 6.1–7.4). Nevertheless, the considerable number of barotrauma cases in our study implies the need for an additional strategy to prevent this complication.

Interestingly, the case group had a longer duration from symptom onset to intubation, and the univariate conditional regression analysis revealed statistical significance (OR, 1.14; CI, 1.006–1.293; *P* = 0.041). This means delayed intubation might induce PNX/PNM. Belletti et al. also identified an extended time from symptom onset to intubation in patients with PNX/PNM, suggesting the harmful effect of delayed intubation [13]. A possible mechanism of these findings is lung injury due to high respiratory drive and large tidal volume in non-intubated patients [21].

Several studies have reported a considerable rate of barotrauma in patients on IMV support and with *Pneumocystis jirovecii* pneumonia (PJP) (13–61%) [22]. Consequently, PJP is recognized as a frequent cause of secondary PNX [23]. The rate of barotrauma due to COVID-19 in this study was similar to that reported in previous studies. Therefore, it is necessary to determine whether PNX/PNM has occurred in patients with severe COVID-19.

While PNX/PNM due to COVID-19 has been primarily reported in patients who are on IMV support, a small number patients who are not on IMV support have also been found to develop PNX/PNM [24, 25]. Despite a lower prevalence of PNX/PNM in patients who are not on IMV support, 6 of 233 with NIAID-OS scores of 5–6 developed PNX/PNM in our study. This appears to be a unique feature of the COVID-19 pandemic. Several studies have suggested alveolar rupture due to severe diffuse alveolar damage and increased intrathoracic pressure by cough or Valsalva maneuver as the mechanisms underlying the development of PNX/PNM among non-ventilated patients with COVID-19 [26, 27].

In our study, for patients with PNX/PNM, the median duration from symptom onset to PNX/PNM was 24.5 days (IQR, 18.5–35.25 days), similar to the results of Belletti et al., suggesting that PNX/PNM occurs as a late complication [13]. Hence, careful observation is necessary even after an acute exacerbation period in COVID-19 management.

The distinct feature of this study is the analysis of the risk factors of PNX/PNM in patients with COVID-19 using a severity-matched case–control group. In addition, since the study period covered the early and recent phases of the COVID-19 pandemic, our results might reflect the general characteristics of COVID-19 rather than the characteristics for a specific period and variant.

This study had several limitations. First, the radiological findings of chest radiography or chest computed tomography could not be analyzed in detail. Second, the number of case patients was small since the data were collected from a single institution. In particular, the lack of clear risk factors with statistical significance in the multivariable analysis could be attributed to the relatively small sample size of our study. Thus, further studies with large sample sizes are needed to clarify the risk factors associated with PNX/PNM in patients with COVID-19.

## Conclusions

In this case–control study, using a control group matched for age, sex, and disease severity, higher BMI tended to show a protective effect against PNX/PNM due to COVID-19, and delayed application of invasive mechanical ventilation was found to be a contributive factor for this complication.

## Data Availability

The datasets used and analysed during the current study are available from the corresponding author on reasonable request.

## Abbreviations

ARDS: Acute respiratory distress syndrome
BMI: Body mass index
CI: Confidence interval
COVID-19: Coronavirus disease-2019
IMV: Invasive mechanical ventilation
IQR: Interquartile range
NIAID-OS: National Institute of Allergy and Infectious Diseases ordinal scale
OR: Odds ratio
PCR: Polymerase chain reaction
PJP: *Pneumocystis jirovecii* Pneumonia
PNM: Pneumomediastinum
PNX: Pneumothorax
SOFA: Sequential organ failure assessment
